# Optimizing retinopathy of prematurity screening in China using a single objective criterion: a 10-year retrospective analysis

**DOI:** 10.3389/fped.2026.1831132

**Published:** 2026-06-17

**Authors:** Zhenglin Li, Suo Guo, Haojue Xu, Wentao Dong

**Affiliations:** Department of Ophthalmology, Sichuan Provincial People’s Hospital, School of Medicine, University of Electronic Science and Technology of China, Chengdu, China

**Keywords:** birth weight, gestational age, over-screening, retinopathy of prematurity, screening criteria

## Abstract

**Objective:**

To evaluate the efficacy of a single objective screening criterion—birth weight (BW) < 2000g or gestational age (GA) < 32 weeks—for retinopathy of prematurity (ROP) detection, and to provide evidence for reducing over-screening caused by subjective clinical judgment.

**Methods:**

This retrospective study enrolled 1,453 premature infants who underwent ROP screening at Sichuan Provincial People's Hospital (2014–2024). Fundus examinations were performed using binocular indirect ophthalmoscopy or RetCam III. A two-tier grouping strategy was adopted: objective criterion group (BW < 2000g or GA < 32 weeks) vs. subjective group (BW ≥ 2000g and GA ≥ 32 weeks). The objective group was further subdivided into three subgroups. ROP and severe ROP were diagnosed following *Chinese Guidelines for the Screening of Retinopathy of Prematurity (2014)*, with severe ROP defined as type 1 ROP requiring clinical treatment. The study population was stratified into inborn infants and referred infants for sensitivity analysis. Multivariable logistic regression identified independent risk factors for severe ROP.

**Results:**

Among 1,453 infants (618 inborn, 836 referred), 705 ROP cases (48.5%) and 370 severe ROP cases (25.5%) were identified. The objective group covered 98.2% of ROP cases and 99.2% of severe ROP cases. ROP and severe ROP prevalence were significantly higher in the objective group than in the subjective group (both *P* < 0.001). Multivariable regression identified lower GA (OR = 1.42 per week decrease) and lower BW (OR = 1.21 per 100 g decrease) as independent risk factors for severe ROP (both *P* < 0.001); sex, case source, and birth year were not significant. All three severe ROP cases in the subjective group were diagnosed in or before 2017, with no new cases reported after 2017.

**Conclusion:**

The single objective criterion of BW < 2000g or GA < 32 weeks can effectively identify the high-risk population for ROP. Subjective extended screening has minimal clinical value in tertiary neonatal care institutions; it is recommended that the subjective high-risk clause may be cautiously omitted in clinical practice within tertiary hospitals with advanced neonatal care capabilities.

## 1 Introduction

Retinopathy of prematurity (ROP) remains a leading cause of preventable childhood blindness, which underscores the urgent need for efficient and accurate screening strategies. The current *Chinese Guidelines for the Screening of Retinopathy of Prematurity (2014)* (1) adopt a dual-entry screening model: (1) objective criteria (BW < 2000g or GA < 32 weeks); and (2) subjective criteria (infants with severe systemic conditions or a documented history of prolonged oxygen exposure, deemed as high-risk by neonatologists). However, the second criterion lacks clear operational definitions, rendering its clinical application heavily dependent on clinicians’ experience and risk perception. In practice, this often leads to the overexpansion of screening scope due to clinical defensive practice, exposing a substantial number of infants who do not meet the objective criteria to repeated fundus examinations.

Such subjective extended screening not only burdens medical resources but also increases psychological stress and financial costs for families, while subjecting infants to unnecessary procedural risks. Given the constraints of healthcare resources and the rising survival rates of preterm infants, optimizing ROP screening to balance diagnostic sensitivity and the avoidance of over-screening has become an urgent clinical priority. Against this backdrop, this large-scale 10-year retrospective study was conducted to evaluate the diagnostic performance of strictly applying the single objective criterion (BW < 2000g or GA < 32 weeks) in detecting ROP and severe ROP. By comparing the screening outcomes with those of the subjective extended screening population, we quantitatively assessed the clinical utility of the subjective high-risk clause, providing evidence to inform the revision of future clinical guidelines.

## 2 Materials and methods

### 2.1 General information

This retrospective study included 1,453 newborns who underwent ROP screening at Sichuan Provincial People's Hospital from January 2014 to March 2024, among whom a considerable proportion were referred ROP cases from secondary hospitals in Sichuan Province. The cohort was stratified into inborn infants (*n* = 618) and referred infants (*n* = 836) for subsequent sensitivity analysis. The cohort comprised 802 males and 651 females, with a median BW of 1430 g (interquartile range, IQR: 1150–1800) and a median GA of 30.9 weeks (IQR: 28.9–32.9). The study was conducted in accordance with the Declaration of Helsinki and approved by the Medical Ethics Committee of Sichuan Provincial People's Hospital, which waived the requirement for written informed consent (Ethics No.: 2025-946).

### 2.2 Methods

#### 2.2.1 Inclusion and exclusion criteria

Inclusion criteria: Newborns who met the criteria of the *Chinese Guidelines for the Screening of Retinopathy of Prematurity (2014)* (1) and underwent ROP screening during the study period, including: (1) preterm or low-birth-weight infants with BW < 2000g or GA < 32 weeks; (2) infants considered as high-risk by pediatricians due to severe illness or a clear history of prolonged oxygen therapy. In this study, all infants meeting the second criterion had BW ≥ 2000g and GA ≥ 32 weeks, representing the sole population subject to extended screening based on subjective pediatric assessment.

Exclusion criteria: (1) Incomplete clinical data; (2) Failure to follow up strictly in accordance with the guideline requirements until complete retinal vascularization or lesion regression due to various reasons; (3) Complicated with other ocular diseases that may affect visual acuity.

#### 2.2.2 Examination

All participants underwent fundus examination after pupillary dilation using binocular indirect ophthalmoscopy or a wide-field digital pediatric retinal imaging system (RetCam III). Clinical data including BW, GA, sex and fundoscopic findings were recorded in detail. Fundus examinations and ROP diagnoses were performed by two senior pediatric retinal specialists in accordance with the *Chinese Guidelines for the Screening of Retinopathy of Prematurity* (2014) (1), and interobserver agreement for ROP diagnosis was assessed using the Kappa coefficient (Kappa > 0.8, indicating excellent consistency). The most severe finding during the entire screening and follow-up period was recorded; for asymmetric bilateral disease, the more severe eye was used for clinical classification. Treatment decisions were made jointly by the two specialists. Severe ROP was defined as type 1 ROP requiring treatment, including zone I stage 1+, 2+, 3 or 3+, and zone II stage 2+ or 3+ .

#### 2.2.3 Grouping

Subjects were first divided into two groups according to screening criteria: (1) Objective screening criterion group: met the objective index criteria (BW < 2000g or GA < 32 weeks), representing the mandatory screening population; (2) Subjective screening criterion group: met only the subjective judgment criteria (BW ≥ 2000g and GA ≥ 32 weeks). The objective group was further subdivided into three subgroups: Term low-birth-weight subgroup (BW < 2000g and GA ≥ 32 weeks); Preterm normal-birth-weight subgroup (BW ≥ 2000g and GA < 32 weeks); Preterm low-birth-weight subgroup (BW < 2000g and GA < 32 weeks). The entire study population was additionally stratified into inborn and referred cases for sensitivity analysis to address potential selection bias from the single-center referral-enriched design.

We calculated the prevalence of ROP and severe ROP in each group and the screening coverage rate of the objective criterion, and analyzed the differences in disease prevalence among the three objective subgroups.

### 2.3 Statistical Analysis

Data were analyzed using SPSS 26.0 and validated with R 4.4.0. The Shapiro–Wilk test was used for the normality test of continuous variables. Variables conforming to the normal distribution were described as mean ± standard deviation (x ± s), and those not conforming to the normal distribution were described as median (lower quartile, upper quartile); the non-parametric Kruskal–Wallis H test was used for intergroup comparison, and Dunn's method was used for pairwise multiple comparison. Categorical variables were described as case number (constituent ratio, %), and the *χ*^2^ test or Fisher's exact test was used for intergroup comparison, with the chi-square partition method for multiple comparison. The test level *α* = 0.05, and the Bonferroni method was used to correct the test level for multiple comparison, with the adjusted significance level set at *α* = 0.017. Multivariable logistic regression analysis was performed to identify independent risk factors for severe ROP, with BW, GA, sex, case source (inborn vs. referred), and birth year included as independent variables; odds ratios (OR) and 95% CIs were calculated for each variable.

## 3 Results

### 3.1 Prevalence of ROP and severe ROP by screening criteria

A total of 705 ROP cases were identified, accounting for 48.5% (705/1453, 95% CI: 45.9–51.1%) of the study cohort. Among these, 692 cases were in the objective screening criterion group (54.8%, 692/1262, 95% CI: 52.0–57.6%) and 13 cases in the subjective screening criterion group (6.8%, 13/191, 95% CI: 3.8–11.4%). The objective criterion covered 98.2% (692/705, 95% CI: 96.8–99.0%) of all ROP cases, and the prevalence of ROP was significantly higher in the objective group than in the subjective group (*χ*^2^ = 198.36, *P* < 0.001).

**Figure 1:**
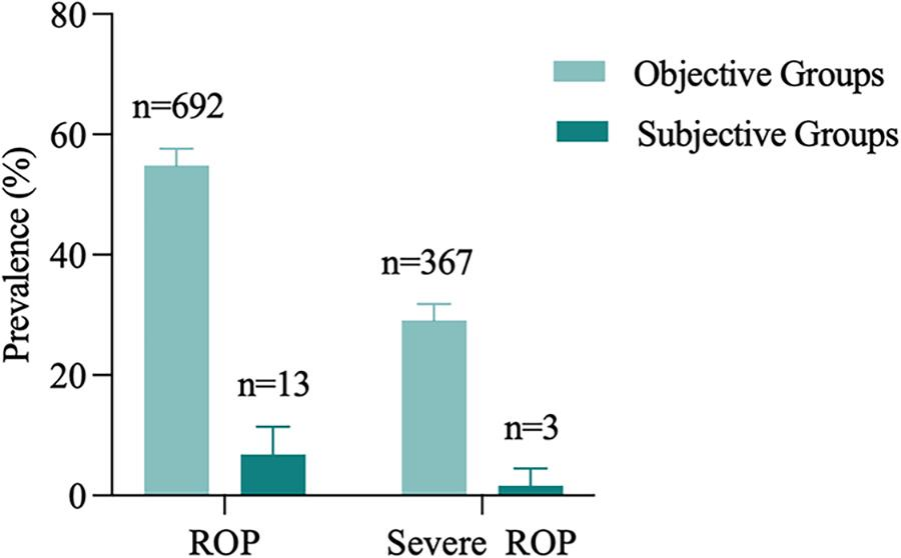
Comparison of ROP and severe ROP prevalence between the objective and subjective groups. Light teal bars represent the objective group, and dark teal bars the subjective group. Error bars indicate 95% confidence intervals. The prevalence of both any ROP and severe ROP was significantly higher in the objective group than in the subjective group (for any ROP: 54.8% vs. 6.8%, *χ*^2^ = 198.36, *P* < 0.001; for severe ROP: 29.1% vs. 1.6%, *χ*^2^ = 127.52, *P* < 0.001).

Severe ROP was diagnosed in 370 infants (25.5%, 370/1453, 95% CI: 23.2–27.8%), with 367 cases in the objective group (29.1%, 367/1262, 95% CI: 26.5–31.8%) and 3 cases in the subjective group (1.6%, 3/191, 95% CI: 0.3%–4.5%). The objective criterion achieved a coverage rate of 99.2% (367/370, 95% CI: 97.6–99.8%) for severe ROP, and the prevalence of severe ROP was also significantly higher in the objective group (*χ*^2^ = 127.52, *P* < 0.001). The comparison is visualized in Figure 1.

Sensitivity analysis for inborn (*n* = 618) vs. referred (*n* = 836) cases showed no significant difference in the screening coverage of the objective criterion between the two subgroups (all *P* > 0.05). The objective criterion covered 97.8% of ROP cases and 98.9% of severe ROP cases in inborn infants, and 98.5% of ROP cases and 99.4% of severe ROP cases in referred infants. The prevalence of ROP and severe ROP was also not significantly different between inborn and referred cases within the objective group (ROP: 53.9% vs. 55.6%, *P* = 0.52; severe ROP: 28.4% vs. 29.7%, *P* = 0.58) (Supplementary Table 1).

### 3.2 Prevalence of ROP and severe ROP in objective subgroups

There were significant differences in the prevalence of ROP and severe ROP among the three subgroups of the objective group (all *P* < 0.001). The preterm low-birth-weight subgroup exhibited the highest ROP incidence (66.4%, 95% CI: 63.2–69.4%) and severe ROP incidence (37.0%, 95% CI: 33.9–40.2%), followed by the preterm normal-birth-weight subgroup (52.2%, 95% CI: 30.0–73.9% and 43.5%, 95% CI: 21.9–66.9%, respectively). Notably, the preterm normal-birth-weight subgroup had a slightly higher severe ROP incidence than the preterm low-birth-weight subgroup, which may be related to its small sample size (*n* = 23). The term low-birth-weight subgroup had the lowest rates of ROP and severe ROP (21.0%, 95% CI: 16.5–26.2% and 4.8%, 95% CI: 2.7%–7.9%, respectively).

There were statistically significant differences in the median BW and GA across the three objective subgroups (*P* < 0.001). The preterm low-birth-weight subgroup had the lowest BW and GA, the term low-birth-weight subgroup had the highest GA, and the preterm normal-birth-weight subgroup had the highest BW (Table 1).

**Table 1:** Comparison of ROP and severe ROP prevalence across study groups.

Group	Screened cases (n, %)	Median BW (g) (IQR)	Median GA (weeks) (IQR)	ROP cases (n, %) (95% CI)	Severe ROP cases (n, %) (95% CI)	*P* value
Objective screening criterion group	1262 (86.9)	1350.0 (1080.0–1750.0)	29.6 (28.0–32.0)	692 (54.8) (52.0–57.6)	367 (29.1) (26.5–31.8)	<0.001
Term low-birth-weight subgroup	314 (21.6)	1717.5 (1480.0–1854.5)	33.0 (32.3–34.0)	66 (21.0) (16.5–26.2)	15 (4.8) (2.7–7.9)	<0.001
Preterm normal-birth-weight subgroup	23 (1.6)	2000.0 (2000.0–2100.0)	31.3 (31.0–31.6)	12 (52.2) (30.0–73.9)	10 (43.5) (21.9–66.9)	<0.001
Preterm low-birth-weight subgroup	925 (63.7)	1270.0 (1050.0–1450.0)	29.0 (28.0–30.4)	614 (66.4) (63.2–69.4)	342 (37.0) (33.9–40.2)	<0.001
Subjective screening criterion group	191 (13.1)	2200.0 (2075.0–2310.0)	34.0 (33.2–35.0)	13 (6.8) (3.8–11.4)	3 (1.6) (0.3–4.5)	–

Comparisons between the objective and subjective groups and among the three objective subgroups were performed using the *χ*^2^ test (all *P* < 0.001). Bonferroni correction set the significance level at *α* = 0.017 for multiple comparisons. BW, birth weight; GA, gestational age; IQR, interquartile range; CI, confidence interval.

### 3.3 BW and GA characteristics of ROP and severe ROP patients

Among all ROP patients, the median BW was 1220 g (IQR: 1000–1450) and the median GA was 29.0 weeks (IQR: 27.9–30.0). For severe ROP patients, the median BW was 1145 g (IQR: 950–1350) and the median GA was 28.0 weeks (IQR: 27.0–29.0). For both ROP and severe ROP patients, BW and GA were significantly lower in the objective subgroups than in the subjective screening group (*P* < 0.001), with the preterm low-birth-weight subgroup having the lowest values and the subjective screening group having the highest (Table 2).

**Table 2:** Birth weight and gestational age of ROP and severe ROP patients by group.

Group	ROP cases (n)	Median BW of ROP (g) (IQR)	Median GA of ROP (weeks) (IQR)	Severe ROP cases (n)	Median BW of severe ROP (g) (IQR)	Median GA of severe ROP (weeks) (IQR)	*P* value
Term low-birth-weight subgroup	66	1630.0 (1435.0–1837.5)	32.9 (32.0–33.6)	15	1650.0 (1425.0–1825.0)	33.0 (32.0–33.0)	<0.001
Preterm normal-birth-weight subgroup	12	2000.0 (2000.0–2000.0)	31.0 (31.0–31.6)	10	2000.0 (2000.0–2000.0)	31.0 (31.0–31.6)	<0.001
Preterm low-birth-weight subgroup	614	1160.0 (980.0–1380.0)	28.7 (27.1–29.9)	342	1145.0 (950.0–1350.0)	28.0 (27.0–29.0)	<0.001
Subjective screening criterion group	13	2150.0 (2090.0–2450.0)	34.3 (33.4–35.0)	3	2150.0 (2120.0–2175.0)	33.0 (32.5–33.5)	<0.001

BW and GA comparisons across groups were performed using the Kruskal–Wallis H test (all *P* < 0.05), with Dunn's test and Bonferroni correction (*α* = 0.017). BW, birth weight; GA, gestational age.

### 3.4 Clinical characteristics of severe ROP patients in the subjective screening group

All three infants with severe ROP in the subjective screening group received intravitreal ranibizumab (IVR) therapy, and all achieved complete retinal vascularization after treatment with no severe visual impairment at the last follow-up.

All severe cases were diagnosed in or before 2017, with no new severe ROP cases identified in the subjective group after 2017. Among them, two cases involved bilateral disease, and one case was unilateral. The detailed clinical characteristics are presented in Table 3. A formal 10-year time-trend analysis (2014–2023) based on our published study confirmed a continuous downward trend in severe ROP incidence in the subjective group, with no severe ROP cases recorded after 2017 (2). Annual numbers of screened infants and severe ROP cases in the subjective extended screening group during 2014–2024 are presented in Supplementary Table 2.

**Table 3:** Clinical characteristics of 3 severe ROP patients in the subjective screening criterion group.

Case	Birth weight (g)	Gestational age (weeks)	Affected eye(s)	Treatment method	Year of treatment
1	2200	32	Both eyes	IVR	2014
2	2150	34	Right eye	IVR	2015
3	2090	33	Both eyes	IVR	2017

IVR, intravitreal injection of ranibizumab.

### 3.5 Independent risk factors for severe ROP

Multivariable logistic regression analysis was performed to identify independent risk factors for severe ROP, with BW (per 100 g decrease), GA (per week decrease), sex (male vs. female), case source (inborn *n* = 618 vs. referred *n* = 836), and birth year as independent variables. The model showed good fit (Hosmer-Lemeshow test: *χ*^2^ = 7.24, *P* = 0.51). Lower GA (per week decrease) was a significant independent risk factor for severe ROP (OR = 1.42, 95% CI: 1.28–1.58, *P* < 0.001), as was lower BW (per 100 g decrease) (OR = 1.21, 95% CI: 1.12–1.31, *P* < 0.001). Sex (male vs. female: OR = 1.08, 95% CI: 0.82–1.42, *P* = 0.59), case source (referred vs. inborn: OR = 1.07, 95% CI: 0.80–1.43, *P* = 0.65), and birth year (per year increase: OR = 0.97, 95% CI: 0.92–1.02, *P* = 0.28) were not significant independent predictors of severe ROP (Table 4). These results confirm that BW and GA are the core independent risk factors for severe ROP in this cohort.

**Table 4:** Multivariable logistic regression analysis of independent risk factors for severe ROP.

Variable	Odds Ratio (OR)	95% Confidence Interval (CI)	*P* value
Gestational age (per week decrease)	1.42	1.28–1.58	<0.001
Birth weight (per 100 g decrease)	1.21	1.12–1.31	<0.001
Sex (Male vs. Female)	1.08	0.82–1.42	0.59
Case source (Referred vs. Inborn)	1.07	0.80–1.43	0.65
Birth year (per year increase)	0.97	0.92–1.02	0.28
Hosmer-Lemeshow goodness-of-fit test	*χ*^2^ = 7.24	–	0.51

## 4 Discussion

Using a two-tier grouping strategy, this study systematically compared the clinical utility of objective and subjective ROP screening criteria in a large sample of preterm infants over a 10-year period. Our findings demonstrate that the single objective criterion (BW < 2000g or GA < 32 weeks) captured 98.2% of all ROP cases and 99.2% of severe ROP cases, thereby identifying nearly the entire high-risk population for ROP, with high screening sensitivity in both inborn and referred cases. In contrast, subjective extended screening detected only a negligible number of ROP cases, with no severe ROP cases identified after 2017; formal time-trend analysis based on our published study confirmed a continuous downward trend in severe ROP incidence in the subjective group (2). These results clearly indicate that subjective extended screening has minimal clinical value in tertiary neonatal care institutions in China under current neonatal care standards and support the tentative exclusion of this clause in clinical practice within tertiary hospitals with advanced neonatal care capabilities. For primary or secondary medical institutions or regions with less advanced neonatal care, the dual criteria should be temporarily retained to avoid missing potential high-risk cases, with gradual adjustments as medical care levels improve.

Low BW and low GA are well-established risk factors for ROP development and show a strong correlation with treatment-requiring severe ROP, which forms the solid pathophysiological basis for the current ROP screening strategies (3, 4). Our multivariable logistic regression analysis further validates that low BW and low GA are the only independent risk factors for severe ROP in this cohort, with no significant contribution from sex, case source, or birth year (all *P* > 0.05). The variability in ROP screening criteria across countries reflects the differences in neonatal care standards, preterm birth rates, survival outcomes and oxygen saturation targets in regions at different developmental stages (5). In the United Kingdom, screening is recommended for infants with BW < 1501 g or GA < 31 weeks (6); in the United States, it is GA ≤ 30 weeks or BW ≤ 1500 g (7). Even within the same country, screening criteria undergo periodic revisions (typically every 5–10 years) in response to evolving epidemiological patterns and advances in neonatal care (8). For instance, the 2008 UK guidelines recommended screening for BW < 1501 g or GA ≤ 31⁺⁶ weeks (9), and the 2022 update lowered the GA threshold to < 31 weeks while retaining the original BW criterion (6). In the United States, most ROP screening programs follow the guidelines of the American Academy of Pediatrics and/or American Academy of Ophthalmology, which have maintained a consistent BW threshold of < 1500 g since 1996, while the GA criterion has varied between 28 and 32 weeks, with the current threshold set at ≤ 30 weeks (10). Similar to China, U.S. guidelines also recommend screening for infants identified as high-risk by neonatologists even if they fall outside the objective criteria (7); however, the clinical effectiveness of this extended screening has not been rigorously validated in large-scale prospective studies. Notably, the 2022 UK ROP guideline has abandoned the risk factor-based screening expansion strategy, which provides an important reference for the revision of ROP screening guidelines in other countries (11).

In this study, the subjective screening criterion group was defined as infants with BW ≥ 2000g and GA ≥ 32 weeks—the population subject to extended screening under the current Chinese guidelines. The observed ROP incidence in this group was 6.8%, with a severe ROP incidence of 0.2%, which is consistent with the results of previous relevant reports (12, 13). Zhang et al. (12) retrospectively analyzed 1,866 infants undergoing ROP screening in Xi’an from 2008 to 2012 and identified ROP in 243 cases (13.0%), including 96 cases (5.1%) of treatment-requiring severe ROP. Among 796 infants who fell outside the BW ≤ 2000g criterion, 24 ROP cases (9.9% of all ROP cases) were detected, including 6 severe ROP cases (6.3% of all severe cases). Luo et al. (13) reviewed 238 preterm infants screened for ROP in Sichuan from 2017 to 2018 and reported ROP in 35 cases (14.7%), with 14 cases (5.9%) requiring treatment. Among the 35 ROP cases, 8 (22.9%) had GA ≥ 32 weeks (accounting for 6.9% of infants in that GA stratum), and 3 (8.6%) had BW ≥ 2000g (accounting for 5.8% of infants in that BW stratum). However, that study did not further analyze the proportion of treatment-requiring cases among ROP patients with GA ≥ 32 weeks or BW ≥ 2000g. Collectively, these findings suggest that extended screening for high-risk infants with severe illness or prolonged oxygen exposure was clinically justified under the earlier versions of ROP screening guidelines. Notably, all three severe ROP cases in our subjective screening group occurred before 2017, and the study by Zhang et al. (12) was conducted prior to 2012. This temporal pattern reflects the substantial improvements in the quality of neonatal intensive care in China over the two decades since the issuance of the *Guidelines for the Clinical Application of Oxygen Therapy and the Prevention and Treatment of Retinopathy of Prematurity in Premature Infants* (14) in 2004, accompanied by a significant declining trend in ROP incidence (15). Unlike ROP screening strategies in many developed countries, which undergo major revisions every 5–10 years, the current Chinese ROP screening criteria have remained basically unchanged since 2004 (14). Consequently, while the existing guidelines maintain high sensitivity for detecting treatment-requiring ROP, they also substantially increase the number of infants requiring routine screening. Whether continued intensive fundus surveillance is warranted for this low-risk population has become a question that warrants further in-depth investigation.

The clinical experience of the Netherlands offers a relevant and valuable precedent for China's revision of ROP screening guidelines. The 2009 NEDROP-1 study revealed that Dutch ROP screening guidelines similarly included a subjective high-risk clause determined by neonatologists (16). In the 2013 guideline revision, the subjective clause was replaced by a tiered strategy: strict objective thresholds (GA < 30 weeks or BW < 1250 g) for mandatory screening, plus five clearly defined objective risk factors (mechanical ventilation, sepsis, necrotizing enterocolitis, postnatal corticosteroids, and hypotension requiring vasoactive drugs) only for infants aged 30–32 weeks or weighing 1250–1500 g, which effectively operationalized the previously subjective criteria (16). The 2017 NEDROP-2 cohort further validated that this refined strategy detected all treatment-requiring ROP cases, reduced the screened population by 23.8%, and cut annual medical costs by €59,454 (16). This experience fully demonstrates that subjective extended screening clauses are not immutable, and can be replaced by more precise and operable objective criteria with the accumulation of clinical evidence. In China, the prevalence of severe ROP in the subjective extended screening population is only 0.2%, with no new cases identified after 2017—an epidemiological profile strikingly similar to that of the Netherlands prior to its guideline revision. This suggests that the evidence base for eliminating the subjective high-risk clause in China's ROP screening guidelines may now be sufficiently established.

The present study confirms that a single screening criterion based solely on BW or GA exhibits high diagnostic sensitivity and good clinical practicality in identifying severe ROP in preterm infants. This finding provides a robust clinical evidence base for optimizing the existing ROP screening criteria in China, particularly in medical institutions with limited screening resources. Importantly, our study differs fundamentally from previous ROP-related studies, which have primarily focused on artificial intelligence (AI)-assisted imaging and diagnostic technologies (17–19). In contrast, the core focus of our research is on the delimitation of the screening population—i.e., who should undergo ROP screening—rather than the technical methods of how to screen. A simplified single-criterion screening approach can substantially improve the efficiency of ROP screening in clinical practice and reduce unnecessary medical interventions and associated risks. To our knowledge, this is among the first large-sample, 10-year retrospective studies conducted in Southwest China that suggests subjective extended screening may no longer identify new cases of severe ROP under modern neonatal care standards, which could further support the clinical value of optimizing screening criteria by eliminating subjective high-risk clauses.

Several limitations of this study should be acknowledged. First, as a single-center retrospective analysis, our cohort included 618 inborn and 836 referred infants; although sensitivity analysis showed no significant difference in screening sensitivity between the two subgroups (*P* > 0.05), potential selection bias cannot be completely excluded. Second, our findings are primarily applicable to tertiary hospitals with advanced neonatal care capabilities, and their generalizability to primary and secondary care settings requires further validation in multicenter studies. Third, detailed clinical variables such as postnatal weight gain and duration of oxygen supplementation were not collected in this study, precluding an in-depth analysis of their potential impact on ROP development in low-risk preterm infants. Fourth, the study did not analyze the interaction between gestational age and birth weight on the incidence of ROP, which may limit the in-depth exploration of the combined risk of the two factors. Future multicenter prospective studies should include diverse populations across different levels of medical care and incorporate comprehensive risk factor analyses (including the interaction of various factors) to inform the formulation of individualized and precise ROP screening strategies.

In conclusion, the single objective criterion (BW < 2000g or GA < 32 weeks) shows high sensitivity and reliability in both inborn and referred infants. Subjective extended screening has minimal clinical value in advanced tertiary neonatal centers and can be cautiously omitted. For lower-level hospitals, the dual criteria may be retained temporarily. This study provides region-specific evidence for optimizing ROP screening in China.

## Data Availability

The datasets presented in this article are not readily available due to patient privacy protection, the raw data cannot be deposited in public repositories. Requests to access the datasets should be directed to the corresponding author upon reasonable request.
