# Supplementary material for: Optimizing retinopathy of prematurity screening in China using a single objective criterion: a 10-year retrospective analysis
Source: Front Pediatr. 2026 Jun 17;14:1831132. doi: 10.3389/fped.2026.1831132 (PMC13319087; doi:10.3389/fped.2026.1831132)
Supplement: Supplementary file 1 [file Table1.docx]

| Supplementary Table 1 Sensitivity analysis of ROP/severe ROP screening coverage by case source (inborn vs. referred) | | | | |
| --- | --- | --- | --- | --- |
| Index | Inborn infants (n=618) | Referred infants (n=836) | χ² value | P value |
| Screened cases meeting objective criterion, n (%) | 602 (85.9) | 660 (87.8) | 0.72 | 0.4 |
| Total ROP cases in subgroup, n | 342 | 363 | – | – |
| ROP cases covered by objective criterion, n (%) (95% CI) | 335 (97.8) (95.9–98.9) | 357 (98.5) (96.8–99.4) | 0.51 | 0.48 |
| Total severe ROP cases in subgroup, n | 172 | 198 | – | – |
| Severe ROP cases covered by objective criterion, n (%) (95% CI) | 170 (98.9) (96.5–99.7) | 197 (99.4) (97.4–99.9) | 0.36 | 0.55 |
| ROP prevalence in objective criterion subgroup, % (95% CI) | 53.9 (50.0–57.8) | 55.6 (52.0–59.2) | 0.32 | 0.57 |
| Severe ROP prevalence in objective criterion subgroup, % (95% CI) | 28.4 (24.8–32.2) | 29.7 (26.2–33.4) | 0.21 | 0.65 |
