# Supplementary material for: Optimizing retinopathy of prematurity screening in China using a single objective criterion: a 10-year retrospective analysis
Source: Front Pediatr. 2026 Jun 17;14:1831132. doi: 10.3389/fped.2026.1831132 (PMC13319087; doi:10.3389/fped.2026.1831132)
Supplement: Supplementary file 2 [file Table2.docx]

| Supplementary Table 2. Annual number of infants screened and severe ROP cases in the subjective extended screening group (birth weight ≥2000 g and gestational age ≥32 weeks), 2014–2024 | | |
| --- | --- | --- |
| Year | Screened infants | Severe ROP (Type 1) |
| 2014 | 20 | 1 |
| 2015 | 19 | 1 |
| 2016 | 20 | 0 |
| 2017 | 21 | 1 |
| 2018 | 19 | 0 |
| 2019 | 18 | 0 |
| 2020 | 17 | 0 |
| 2021 | 16 | 0 |
| 2022 | 16 | 0 |
| 2023 | 15 | 0 |
| 2024 | 10 | 0 |
| Total | 191 | 3 |
